# Ambient Temperature and Cerebrovascular Hemodynamics in the Elderly

**DOI:** 10.1371/journal.pone.0134034

**Published:** 2015-08-10

**Authors:** Wen-Chi Pan, Melissa N. Eliot, Petros Koutrakis, Brent A. Coull, Farzaneh A. Sorond, Gregory A. Wellenius

**Affiliations:** 1 Department of Epidemiology, Brown University School of Public Health, Providence, RI, United States of America; 2 Department of Environmental Health, Harvard School of Public Health, Boston, MA, United States of America; 3 Department of Biostatistics, Harvard School of Public Health, Boston, MA, United States of America; 4 Department of Neurology, Brigham and Women’s Hospital and Institute for Aging Research, Hebrew SeniorLife, Boston, MA, United States of America; McGill University Health Center / Royal Victoria, CANADA

## Abstract

**Background and Purpose:**

Some prior studies have linked ambient temperature with risk of cerebrovascular events. If causal, the pathophysiologic mechanisms underlying this putative association remain unknown. Temperature-related changes in cerebral vascular function may play a role, but this hypothesis has not been previously evaluated.

**Methods:**

We evaluated the association between ambient temperature and cerebral vascular function among 432 participants ≥65 years old from the MOBILIZE Boston Study with data on cerebrovascular blood flow, cerebrovascular resistance, and cerebrovascular reactivity in the middle cerebral artery. We used linear regression models to assess the association of mean ambient temperature in the previous 1 to 28 days with cerebrovascular hemodynamics adjusting for potential confounding factors.

**Results:**

A 10°C increase in the 21-day moving average of ambient temperature was associated with a 10.1% (95% confidence interval [CI], 2.2%, 17.3%) lower blood flow velocity, a 9.0% (95% CI, 0.7%, 18.0%) higher cerebrovascular resistance, and a 15.3% (95%CI, 2.7%, 26.4%) lower cerebral vasoreactivity. Further adjustment for ozone and fine particulate matter (PM_2.5_) did not materially alter the results. However, we found statistically significant interactions between ambient temperature and PM_2.5_ such that the association between temperature and blood flow velocity was attenuated at higher levels of PM_2.5_.

**Conclusions:**

In this elderly population, we found that ambient temperature was negatively associated with cerebral blood flow velocity and cerebrovascular vasoreactivity and positively associated with cerebrovascular resistance. Changes in vascular function may partly underlie the observed associations between ambient temperature and risk of cerebrovascular events.

## Introduction

Ambient temperature has been associated with increased risk of cerebrovascular events and cerebrovascular death [1–9], although results of prior studies have been inconsistent. For example, risk of ischemic stroke has been both positively [3–5] and negatively [6–9] associated with ambient temperature. In the peripheral circulation, higher ambient temperatures have been associated with reduced flow-mediated dilation in the brachial artery [10, 11], a marker of vascular reactivity. However, the effects of ambient temperature on the cerebral circulation have not been previously studied.

The potential effects of ambient temperature on cerebrovascular hemodynamics must be considered in the context of ambient air pollution, which has been repeatedly linked to changes in peripheral vascular function [12–15]. We have previously reported an association between ambient fine particulate matter air pollution (PM_2.5_) and resting cerebrovascular flow and resistance [16]. While that analysis adjusted for potential confounding by ambient temperature, we did not consider the potential interactions between temperature and PM_2.5_ or the potential associations with O_3_.

Understanding the relationships between ambient temperature and cerebrovascular function may yield insights into the mechanisms of weather-related cerebrovascular events and inform future prevention or treatment strategies. Accordingly, we evaluated the association between ambient temperature and cerebrovascular flow, resistance, and vasoreactivity in a prospective cohort of community-dwelling elderly in the Boston metropolitan area. A secondary goal was to evaluate the joint effects on cerebral hemodynamics of temperature with either PM_2.5_ or O_3_.

## Materials and Methods

### Study Design

We evaluated the association between short-term changes in ambient temperature and markers of cerebrovascular hemodynamics among 423 participants in the MOBILIZE Boston study, a prospective, community-based cohort study [17]. Briefly, between 2005 and 2008 we recruited 765 non-institutionalized men and women aged ≧65 years who were able to communicate in English, resided within 5 miles (8.0 km) from the study clinic, and were able to walk 20 feet (6.1 m) without personal assistance. Individuals with a Mini-Mental State Examination score <18 were not eligible to participate. On enrollment, subjects participated in an in-home interview followed within 4 weeks by a clinic examination. We assessed participant characteristics, medical history, medication inventory, smoking history, blood pressure, height, and weight, as previously described [18]. A second in-home interview and clinic examination (follow-up visit) were performed a median of 16.5 months after the baseline visit. All participants provided written informed consent upon enrollment. The study was approved by the Institutional Review Boards at Hebrew Senior Life and Brown University.

Participants were classified as normotensive if blood pressure was <140/90mmHg and there was no history of hypertension or receiving antihypertensive medications; controlled hypertensive if blood pressure was <140/90 mmHg and there was a history of hypertension or receiving antihypertensive medication; and uncontrolled hypertensive if blood pressure was ≥140/90mmHg. A blood sample was collected during the clinic visit and participants were classified as having diabetes mellitus if they reported a past diagnosis of diabetes, they reported using any diabetes medications, were found to have a hemoglobin A1c ≥6.5%, or had a random glucose measurement ≥200 mg/dl. Height and weight were measured during the clinic visit according to a standard protocol and body mass index calculated.

### Cerebrovascular Hemodynamics

At each clinic examination we evaluated participants’ cerebrovascular hemodynamics at rest and during provocative stimulation, as previously described [17]. Briefly, we used transcranial Doppler ultrasound (TCD) to continuously measure cerebral blood flow velocity in the middle cerebral artery (MCA) while participants sat in a chair. A 2-MHz TCD probe (MultiDop X4, DWL-Transcranial Doppler Systems Inc., Sterling, VA) was placed over the right or left temporal bone with the best signal and held in place during recordings using a Velcro headband. TCD data could not be obtained in some participants because of the absence of a suitable acoustic window to insonate the MCA. We measured arterial blood pressure using a Finometer photoplethysmographic system (Finapres Medical Systems, Arnhem, the Netherlands) placed on a finger and held at heart level with a sling. The envelope of the velocity waveform was digitized at 500 Hz, displayed simultaneously with the blood pressure, ECG, and end-tidal CO_2_ signals; and stored for later offline analysis. Cerebrovascular resistance was calculated as the ratio of mean arterial pressure to blood flow velocity [19].

After a 5-minute resting period, we assessed cerebral vasoreactivity by asking participants to breathe room air normally for 2 minutes, inspire a gas mixture of 8% CO_2_, 21% O_2_, and balance N_2_ for 2 minutes, and then mildly hyperventilate to an end-tidal CO_2_ of ≈25 mm Hg for 2 minutes. Cerebral vasoreactivity was calculated as the slope of the linear regression of mean MCA blood flow velocity versus end-tidal CO_2_ during the maneuver.

### Meteorological and Air Pollution Data

We obtained hourly ambient temperature and other meteorological data from the National Weather Service station at Boston’s Logan Airport. PM_2.5_ was measured continuously at the Boston/Harvard ambient monitoring station, as previously described [20]. This monitor was <10 km of the clinic site and <20 km from participants’ residential address. Hourly O_3_ measurements were obtained from the Massachusetts Department of Environmental Protection’s Greater Boston monitoring sites and averaged. For each participant we estimated average ambient temperature, dew point temperature, PM_2.5_, and O_3_ levels in the 1, 2, 3, 5, 7, 14, 21, and 28 days prior to the clinical visit when cerebrovascular hemodynamics were measured.

### Statistical Methods

Of the 765 participants in the MOBILIZE Boston Study, we excluded 76 participants with a history of stroke, 1 participant with a clinical visit on a weekend (making it difficult to statistically adjust for potential day of week effects), and 265 subjects due to the absence of a suitable acoustic window to insonate the MCA, leaving 423 participants for this analysis. Among these 423 participants, TCD data on cerebrovascular hemodynamics were available at two visits in 258 (61%) participants and at one visit in 165 participants.

We used linear mixed effects models with random subject intercepts to assess the association between ambient temperature and cerebrovascular hemodynamics while accounting for repeated measures within individuals. Measures of cerebral hemodynamics (resting blood flow velocity, cerebrovascular resistance, mean arterial pressure, and cerebrovascular reactivity) were all natural log-transformed and results are expressed as the percent difference (and 95% confidence interval [CI]) in each outcome per 10°C change in ambient temperature, 10 μg/m^3^ change in PM_2.5_, or 10 ppb change in O_3_. All models were adjusted for age (natural cubic spline with 3 degrees of freedom), sex, race (white *versus* others), smoking status (never *versus* ever), hypertension status (normotension, controlled hypertension, *versus* uncontrolled hypertension), diabetes mellitus, body mass index (natural cubic spline with 3 degrees of freedom), visit number (baseline *versus* follow-up visit), day of week (indicator variables), seasonal pattern (sine and cosine of calendar time with period of 1 year), and long-term temporal trends (centered time as linear and quadratic functions). Where indicated, we used natural cubic splines with 3 degrees of freedom, resulting in a function with 2 internal knots placed at the upper and lower tertiles of the distribution of the relevant variable. In sensitivity analyses we further adjusted for dew point temperature, PM_2.5_, or O_3_ in separate models. The exposure-response function of each outcome with temperature, PM_2.5_, and O_3_ was initially modeled as a linear function and, subsequently, as a natural cubic spline with 3 degrees of freedom.

In a set of secondary analyses, we evaluated potential interactions between ambient temperature and PM_2.5_ and between ambient temperature and O_3_ by assuming linear exposure-response functions and including interaction terms in our main models. In additional sensitivity analyses, we verified these results using a low rank tensor product smoothing which allows more flexible exposure-response functions for the main effects and 2-way interactions [21]. Analyses were performed using R statistical software (version 3.1.0). A 2-sided *P*-value of <0.05 was considered statistically significant.

## Results

At baseline, the 423 participants with at least one assessment of cerebrovascular hemodynamics were predominantly female (56.0%) and white (86.1%), with a mean age of 77.7 (standard deviation [SD], 5.2) years (Table 1). Over the course of the study period, the mean ambient temperature was 11.0°C (SD: 9.2°C), mean levels of PM_2.5_ were 9.0 μg/m^3^ (SD: 5.3 μg/m^3^), and mean O_3_ levels were 22.9 ppb (SD: 10.7 ppb). Average pollutant levels over the 1 to 28 days prior to each clinical assessment are shown in Table A in S1 Tables. The intraclass correlation coefficients for resting cerebrovascular resistance, mean arterial pressure, and blood flow velocity ranged from 0.81 to 0.84, indicating high within-person reproducibility of these measures over time.

**Table 1:** Characteristics of 423 Participants from the MOBILIZE Boston Study.

Characteristics	
Age, Mean ± SD (years)	77.7 ± 5.2
Female, n (%)	237 (56.0)
White, n (%)	364 (86.1)
Body Mass Index, Mean ± SD	26.5 ± 4.7
Ever Smoker, n (%)	242 (57.2)
Hypertension, n (%)	
Normotension	109 (25.8)
Controlled Hypertension	224 (53.0)
Uncontrolled Hypertension	89 (21.0)
Diabetes Mellitus, n (%)	65 (15.4)
Blood Flow Velocity (cm/s), Mean ± SD	41.8 ± 10.3
Cerebrovascular Resistance (mmHg.s/cm), Mean ± SD	1.76 ± 0.56
Mean Arterial Pressure (mmHg), Mean ± SD	113.3 ± 19.2
Cerebral Vasoreactivity (cm/s.mmHg), Mean ± SD	1.24 ± 0.41

Abbreviations: SD, standard deviation.

**Fig 1:**
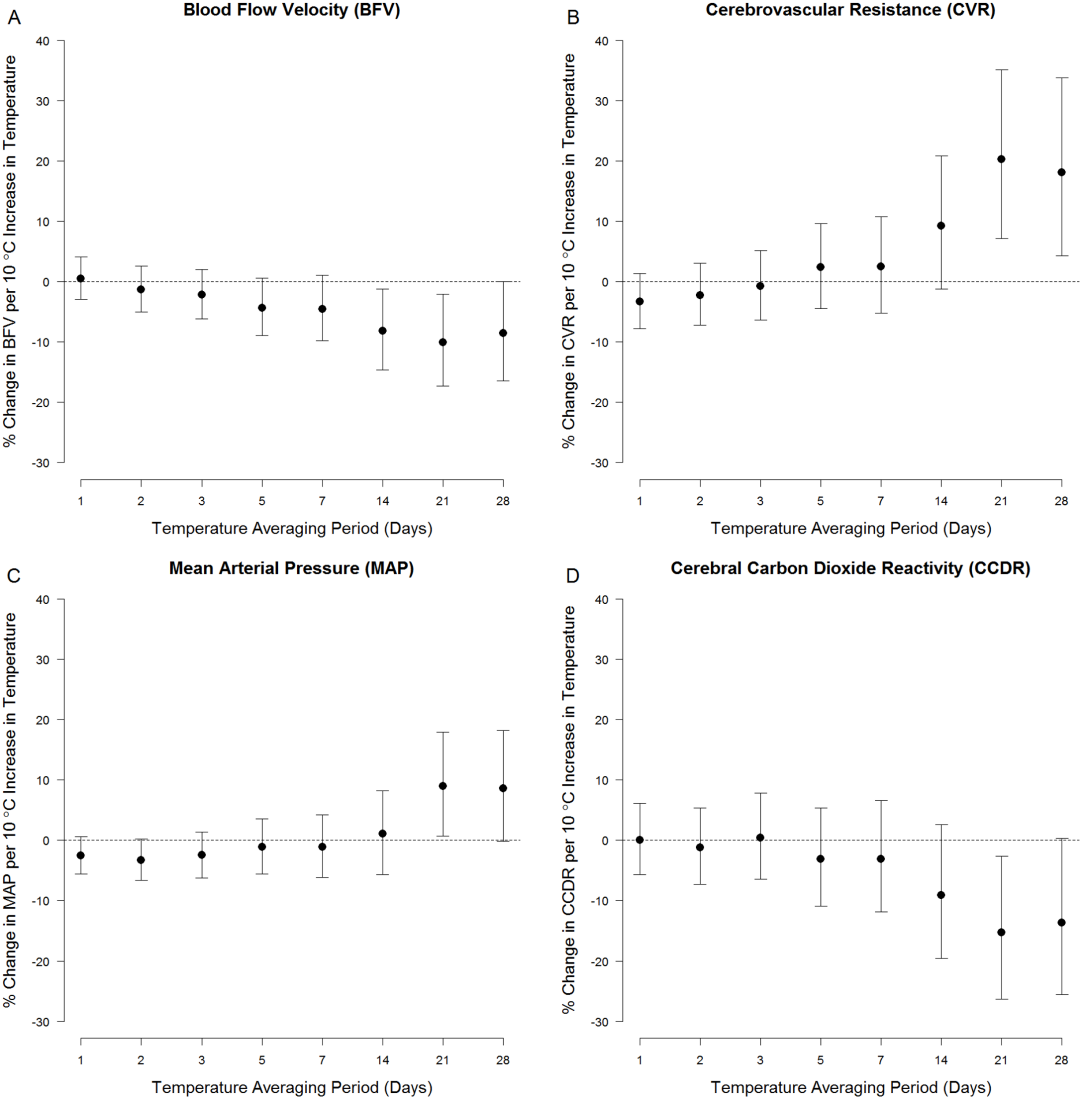
Association between ambient temperature and (A.) resting blood flow velocity (BFV), (B.) resting cerebrovascular resistance (CVR), (C.) resting mean arterial pressure (MAP), (D.) and cerebral varoreactivity (Cerebral VR). The *y*-axis denotes the % change (and 95% confidence interval) in each outcome per 10°C increase in temperature, adjusted for age, sex, race, smoking, hypertension, diabetes, BMI, visit number, day of week, season, and long-term time trends. The x-axis denotes the averaging period for ambient temperature (in days) prior to the TCD assessment.

**Fig 2:**
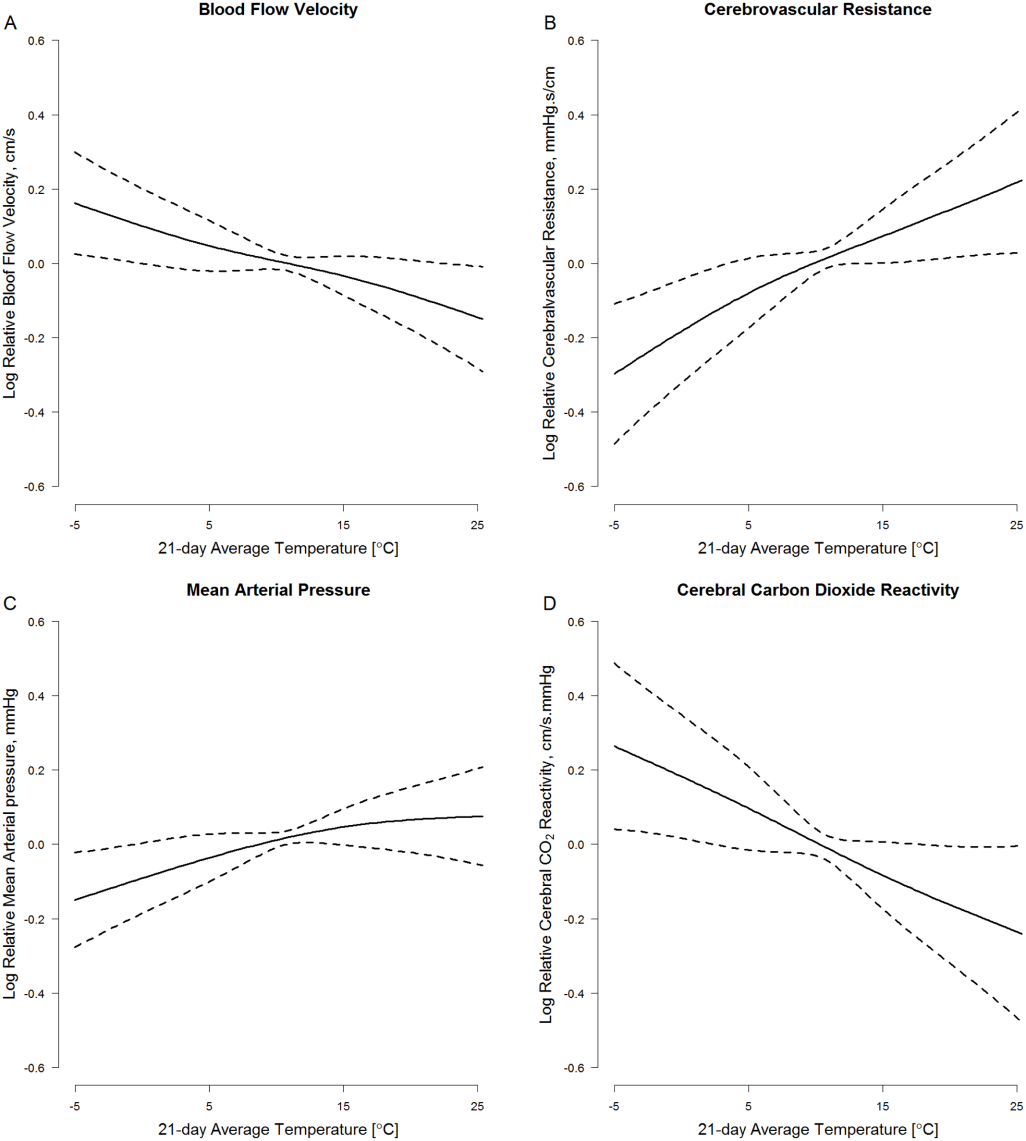
Exposure-response functions between 21-day average ambient temperature and (A.) resting blood flow velocity, (B.) resting cerebrovascular resistance, (C.) resting mean arterial pressure, and (D.) cerebral varoreactivity modeled using natural cubic splines with 3 degrees of freedom. Dashed lines represent 95% confidence intervals.

Measures of cerebral hemodynamics were associated with ambient temperature averaged over longer periods (Fig 1). For example, a 10°C increase in ambient temperature averaged over the prior 21 days (i.e.: the 21-day moving average) was associated with a 10.1% (95% CI: 2.2%, 17.3%) decrease in resting blood flow velocity, a 20.3% (95% CI: 7.1%, 35.1%) increase in resting cerebrovascular resistance, a 9.0% (95% CI: 0.7%, 18.0%) increase in resting mean arterial pressure, and a 15.3% (95%CI: 2.7%, 26.4%) decrease in cerebral vasoreactivity (Table B in S1 Tables, Main Model). We used natural cubic splines and confirmed that the exposure-response functions underlying these observed associations were approximately linear (Fig 2 and S1 Fig). Ambient temperature was not associated with any outcome at shorter moving averages.

In sensitivity analyses, we assessed the robustness of the relationship between ambient temperature and cerebrovascular hemodynamics by further adjusting for PM_2.5_, O_3_, or dew point temperature in separate models (Table B in S1 Tables). Adjustment for PM_2.5_ modestly attenuated the results although the overall pattern of the results remained unchanged. Adjustment for O_3_ had little or no impact on the results. Adjustment for dew point temperature had an inconsistent effect on the results, but substantially increased the width of the confidence intervals suggesting strong colinearity between ambient temperature and dew point (Spearman’s rank correlation coefficients 0.93 to 0.98).

We evaluated the potential interaction between temperature and PM_2.5_ on cerebrovascular hemodynamics and found that PM_2.5_ modified the relationship between temperature and resting blood flow velocity, reaching statistical significance for the 5-, 14-, 21-, and 28-day moving averages (Table 2). Specifically, the association of ambient temperature with blood flow velocity was significantly attenuated at higher PM_2.5_ concentrations and *vice versa*. For example, a 10°C increase in the 21-day moving average of ambient temperature was associated with a 9.0% lower resting blood flow velocity at the 25^th^ percentile of PM_2.5_ (6.76 μg/m^3^), but a 6.4% lower resting blood flow velocity at the 75^th^ percentile of PM_2.5_ (9.85 μg/m^3^) (*P* for interaction = 0.02). We found no statistically significant interactions between ambient temperature and PM_2.5_ on other outcomes. We confirmed these findings using a more flexible 2-way interaction model that showed a similar pattern of results (data not shown). We found no evidence of interaction between ambient temperature and O_3_ with any measure of cerebrovascular function (Table C in S1 Tables).

**Table 2:** Joint effects of ambient temperature and PM_2.5_ on blood flow velocity.

Moving Average (Days prior to clinic visit)	*P* Interaction^a^	% Difference (95% CI) in Blood Flow Velocity per 10°C Increase in Ambient Temperature		% Difference (95% CI) in Blood Flow Velocity per 10 μg/m^3^ Increase in PM_2.5_	
		PM_2.5_ at 25th percentile^b^	PM_2.5_ at 75th percentile^b^	Temperature at 25th percentile^c^	Temperature at 75th percentile^c^
1	0.08	0.1(-4.5, 4.8)	1.4 (-3.1, 6.3)	-4.2 (-10.0, 3.2)	0.3(-8.7, 4.6)
2	0.22	-0.9 (-6.1, 4.6)	0.2 (-5.0, 5.8)	-5.2 (-11.9, 3.1)	-1.7 (-10.9, 4.3)
3	0.10	-1.7 (-7.4, 4.4)	-0.1 (-6.0, 6.0)	-7.3 (-14.0, 1.9)	-2.2 (-12.6, 3.5)
5	0.05	-4.6 (-11.3, 2.7)	-2.7 (-9.5, 4.7)	-8.9 (-16.0, 2.3)	-1.4 (-14.3, 4.3)
7	0.11	-3.5 (-11.2, 4.9)	-1.8 (-9.6, 6.7)	-10.9 (-18.9, 0.7)	-4.6 (-17.6, 2.4)
14	0.02	-7.8 (-17.0, 2.5)	-4.9 (-14.4, 5.7)	-15.7 (-23.6, -1.2)	-3.5 (-21.2, 1.9)
21	0.02	-9.0 (-19.4, 2.7)	-6.4 (-17.0, 5.7)	-19.6 (-27.5, -5.0)	-7.5 (-25.4, -2.2)
28	<0.01	-7.6 (-18.7, 5.0)	-4.2 (-15.7, 8.9)	-22.1 (-29.2, -6.4)	-6.5 (-26.6, -3.0)

Abbreviations: PM_2.5_, fine particulate matter.

^a^ From models including covariates for temperature, PM_2.5_, the cross product of temperature and PM_2.5_, and adjusting for potential confounding factors.

^b^ The 25^th^ percentile of PM_2.5_ at ranged from 5.19 to 6.88 μg/m^3^, and the 75^th^ percentile ranged from 9.93 to 10.2 μg/m^3^.

^c^ The 25^th^ percentile of ambient temperature ranged from 2.81 to 3.38°C, and the 75^th^ percentile ranged from 18.1 to 18.7°C.

## Discussion

In this cohort of elderly participants, we evaluated the association between cerebral hemodynamics and mean ambient temperature in the prior 1 to 28 days and found that, at longer averaging times, higher ambient temperatures were associated with higher mean arterial pressure, higher resting cerebrovascular resistance, lower resting blood flow velocity, and lower cerebrovascular reactivity in response to changing end-tidal CO_2_ levels. Additionally, at longer averaging times we found evidence of an interaction between ambient temperature and PM_2.5_ in association with blood flow velocity.

To our knowledge, this is the first published study designed to evaluate the association between ambient temperature and cerebrovascular hemodynamics. Thus, direct comparison to prior studies is not possible. However, rewarming patients who have moderate hypothermia above 37°C decreases cerebrovascular pressure reactivity index, an indicator for cerebral vascular reactivity [22], suggesting elevated body temperature may perturb cerebrovascular regulation and potentially lead to increased risk of stroke [23].

More is known about the effects of ambient temperature on the peripheral circulation. Nawrot et al [10]. found that higher ambient temperatures in the prior 1 to 21 days were associated with reduced brachial artery flow-mediated vasodilatation, indicative of reduced endothelial function. Similarly, in the Framingham Heart Study, Widlansky *et a*l [11]. found that higher same-day temperatures were associated with reduced hyperemic flow velocity, a marker of peripheral microvascular vasodilator function. These results may be considered analogous to and qualitatively consistent with our findings of reduced cerebrovascular reactivity in the MCA territory in association with ambient temperature, albeit over different time scales. On the other hand, Widlansky *et al*. [11] found no association between same-day temperature and either resting brachial artery diameter or flow-mediated dilation. In a repeated-measures study among patients with type 2 diabetes, Zanobetti *et al*. found that same-day temperature was positively associated with brachial artery diameter but not with either flow-mediated dilation or nitroglycerin-mediated dilation [24]. These studies differ from ours, in part, in the characteristics of participants and time periods considered. Importantly, only the study by Nawrot et al. [10] considered the effects of averages of temperature longer than 5 days on markers of endothelial function.

We have previously reported that in this cohort PM_2.5_ is associated with higher resting cerebrovascular resistance and lower resting blood flow velocity at longer moving averages [16]. In the current study we found evidence of an interaction between PM_2.5_ and ambient temperature for blood flow velocity, but not other outcomes. The physiologic basis for this interaction is unclear, but since the observed associations with temperature and PM_2.5_ are in the same direction (i.e.: to decrease blood flow velocity), it is plausible that as blood flow velocity decreases other compensatory mechanisms are activated to preserve cerebral blood flow. Nonetheless, if our findings on cerebral circulation can be extrapolated to the peripheral circulation, our results suggest that there may be important interactions between ambient temperature and at least some ambient air pollutants in eliciting endothelial dysfunction, but these interactions might only be observed at longer averaging times.

Arterial blood pressure has been shown to be negatively associated with same-day ambient temperature [25–29]. We similarly observed a modest negative association between mean arterial pressure and ambient temperature averaged over the prior 1–3 days, although these results did not reach statistical significance. However, at longer averaging periods, we found that ambient temperature was positively and significantly associated with mean arterial pressure. Few prior studies have considered comparably longer averaging periods. However, previous authors report that nighttime temperature is positively associated with blood pressure, potentially via reduced sleep quality or duration [30, 31], suggesting that temperature may affect blood pressure via different physiologic mechanisms depending on both the time frame and the context of exposure. Our findings that the association between temperature and arterial blood pressure may be in opposite directions depending on the time frame considered raises potentially interesting questions about the differential effects of temperature on the risk of cardiovascular events. Indeed, time-series studies have often found that excess heat is associated with increased risk of cardiovascular events over the next 1–3 days while excess cold increases the risk of cardiovascular events over the next month or so [32, 33]. Additional studies confirming or refuting our observations are clearly needed.

Impaired vasoreactivity assessed by TCD is an established predictor of subsequent stroke, especially among patients with high grade carotid artery disease [34]. If higher temperatures do indeed increase the risk of cererbrovascular events (although, as mentioned above, the evidence for this remains equivocal), our results implicate impaired vasoreactivity as one potential mechanism.

We found no association between O_3_ and any measure of cerebrovascular hemodynamics. This finding is consistent with previous experimental and observational studies failing to find associations between recent O_3_ levels and peripheral vascular function [29, 35, 36]. Additionally, our findings indicated that the association between ambient temperature and blood flow velocity was modified by PM_2.5_ levels. Although we are not aware of any studies examining how air pollutants interact with temperature on cerebrovascular function, several observational studies suggest the interplay of meteorological variables and air pollutants on peripheral vascular function. For example, Hampel *et al*. [37] found a significant interaction between PM_2.5_ and ambient temperature on blood pressure among pregnant women. Further experimental studies may be needed to uncover the biological mechanism underlying the potentially complex interactions between pollutants and meteorological variables in eliciting vascular responses.

This study has a number of potential limitations. First, the high correlation between ambient temperature and dew point in this dataset makes it difficult to separate the effects of these exposures. Second, the 423 subjects included in this analysis tended to be leaner, younger, less likely to have hypertension, and more likely to be white compared to the overall cohort population [19]. Therefore, our findings may not be generalizable to the entire MOBILIZE Boston Study cohort or the general elderly population. Moreover, our results may not be generalizable to populations in other geographic areas with different climatic conditions as well as different levels and sources of air pollution. Third, as has been done in past studies, we used ambient temperature data from a single nearby weather station, potentially leading to some exposure misclassification. More importantly, ambient temperature may not accurately reflect actual temperature exposures during times when participants are indoors. Indoor or personal temperature exposure may have different effects on cerebrovascular hemodynamics, but we were unable to explore this possibility due to the lack of information on participants’ indoor environment. Fourth, our results reflect a combination of the associations observed among repeated measures within the same individual and cross-sectional associations observed across individuals. On the other hand, our study has several strengths including a novel hypothesis, a relatively large sample size, a well-characterized study population, and a detailed assessment of cerebrovascular hemodynamics.

## Conclusions

In conclusion, in this cohort of elderly participants, we found that ambient temperature was associated with lower cerebral blood flow velocity, higher cerebrovascular resistance, higher mean arterial blood pressure, and lower cerebral vasoreactivity. The association between ambient temperature and blood flow velocity was attenuated at high levels of PM_2.5_, but unaffected by ambient O_3_ levels. These findings build upon and extend the growing literature on peripheral vascular effects of temperature and air pollution and provide insights into the potential mechanisms of weather-related cardiovascular morbidity and mortality.

## Supporting Information

S1 Fig Exposure-response functions between ambient temperature and markers of cerebral hemodynamics among 423 participants in the MOBILIZE Boston Study. Natural cubic splines with 3 degrees of freedom were applied to model the association between ambient temperature and each outcome (blood flow velocity, cerebrovascular resistance, mean arterial pressure, and cerebral vasoreactivity) averaging temperature over different periods (1-, 7-, 14-, 21-, or 28-day) prior to the clinic visit. The dashed lines denote the 95% confidence intervals. The carpet plots along the *x*-axis denote the density of temperature values. All models were adjusted for age, sex, race, smoking status, hypertension status, diabetes, body mass index, visit number, day of week, season, and long-term temporal trends. The concentration-response plot of 1-day moving average was similar to the 2- and 3-day plots, and the 7-day moving average plot was similar approximate to the 5-day plot.
(TIFF)

S1 Tables Supplemental tables. (DOCX)
