# Supplementary figures and images for: Ambient Temperature and Cerebrovascular Hemodynamics in the Elderly
Source: PLoS One. 2015 Aug 10;10(8):e0134034. doi: 10.1371/journal.pone.0134034 (PMC4721538; doi:10.1371/journal.pone.0134034)

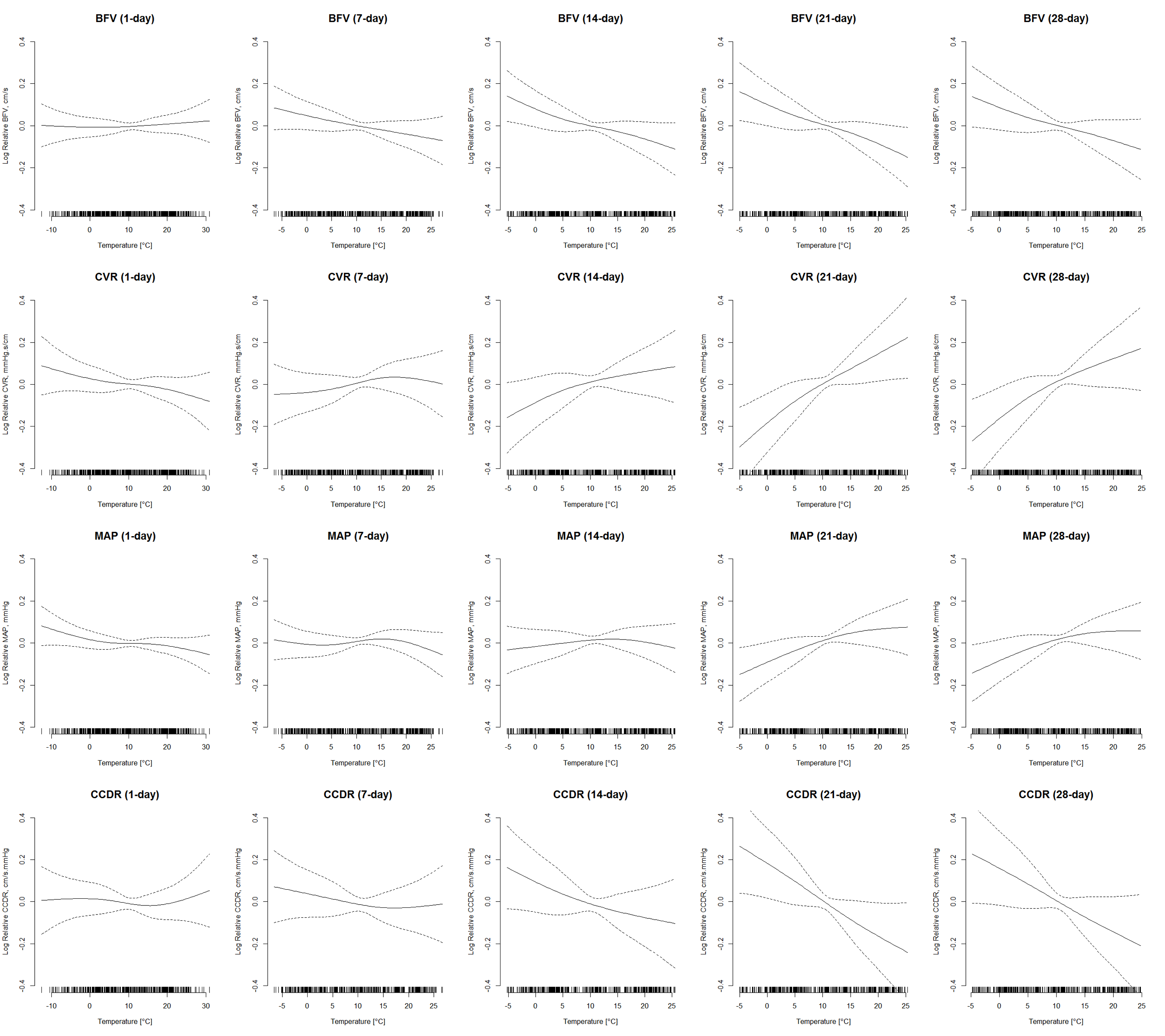

Supplement: S1 Fig — Natural cubic splines with 3 degrees of freedom were applied to model the association between ambient temperature and each outcome (blood flow velocity, cerebrovascular resistance, mean arterial pressure, and cerebral vasoreactivity) averaging temperature over different periods (1-, 7-, 14-, 21-, or 28-day) prior to the clinic visit. The dashed lines denote the 95% confidence intervals. The carpet plots along the x-axis denote the density of temperature values. All models were adjusted for age, sex, race, smoking status, hypertension status, diabetes, body mass index, visit number, day of week, season, and long-term temporal trends. The concentration-response plot of 1-day moving average was similar to the 2- and 3-day plots, and the 7-day moving average plot was similar approximate to the 5-day plot. (TIFF) [file pone.0134034.s001.tiff]
