# Supplementary material for: Ambient Temperature and Cerebrovascular Hemodynamics in the Elderly
Source: PLoS One. 2015 Aug 10;10(8):e0134034. doi: 10.1371/journal.pone.0134034 (PMC4721538; doi:10.1371/journal.pone.0134034)
Supplement: S1 Tables — (DOCX) [file pone.0134034.s002.docx]

Supplemental Tables for:

**Ambient Temperature and Cerebrovascular Hemodynamics in the Elderly**

Wen-Chi Pan, Melissa N. Eliot, Petros Koutrakis, Brent A. Coull, Farzaneh A. Sorond, and Gregory A. Wellenius

From the Department of Epidemiology, Brown University, Providence, RI (W-C.P., M.N.E., G.A.W.), the Departments of Environmental Health (P.K.) and Biostatistics (B.A.C.), Harvard School of Public Health, Boston, MA, Department of Neurology, Brigham and Women’s Hospital and Institute for Aging Research, Hebrew SeniorLife, Boston, MA (F.A.S.)

**Address correspondence to:** Gregory A. Wellenius, Associate Professor of Epidemiology, Brown University, Box G-121S, Providence, RI 02912 USA. Telephone: (401) 863-9649. Fax: (401) 863­3713. E-mail: [gwelleni@brown.edu](mailto:gwelleni@brown.edu)

**Table A.** Summary statistics for ambient temperature, PM_2.5_, and ozone for different averaging periods prior to the clinic visit.

| **Moving Average, Day** | **Temperature, °C** | | | |  | **PM_2.5_, μg/m^3^** | | | |  | **Ozone, ppb** | | | |
| --- | --- | --- | --- | --- | --- | --- | --- | --- | --- | --- | --- | --- | --- | --- |
|  | Mean | SD | Median | IQR |  | Mean | SD | Median | IQR |  | Mean | SD | Median | IQR |
| 1 | 10.6 | 9.4 | 10.9 | 15.4 |  | 8.4 | 4.8 | 7.0 | 4.7 |  | 23.8 | 10.6 | 22.6 | 14.4 |
| 2 | 10.7 | 9.3 | 10.6 | 15.4 |  | 8.5 | 4.2 | 7.2 | 4.7 |  | 24.4 | 9.7 | 23.6 | 13.5 |
| 3 | 10.7 | 9.1 | 10.8 | 15.3 |  | 8.5 | 3.9 | 7.3 | 4.5 |  | 24.4 | 9.1 | 23.6 | 13.4 |
| 5 | 10.6 | 8.9 | 10.5 | 15.3 |  | 8.4 | 3.2 | 7.4 | 3.8 |  | 24.2 | 8.3 | 23.2 | 12.8 |
| 7 | 10.7 | 8.7 | 10.5 | 14.9 |  | 8.5 | 3.0 | 7.6 | 3.7 |  | 24.3 | 7.9 | 24.3 | 13.5 |
| 14 | 10.8 | 8.5 | 10.6 | 15.2 |  | 8.6 | 2.6 | 7.9 | 3.5 |  | 24.3 | 7.5 | 24.5 | 13.2 |
| 21 | 10.8 | 8.4 | 10.6 | 15.2 |  | 8.6 | 2.3 | 8.0 | 3.1 |  | 24.3 | 7.2 | 24.6 | 13.3 |
| 28 | 10.8 | 8.3 | 10.8 | 15.3 |  | 8.6 | 2.2 | 8.1 | 3.0 |  | 24.3 | 7.1 | 24.5 | 12.8 |

Abbreviations: SD, standard deviation; IQR, interquartile range.

**Table B.** Association between ambient temperature and markers of cerebral hemodynamics, with and without adjustment for PM_2.5_, ozone, and dew point temperature.

| **Moving Average, Day** | **% Difference in Cerebral Hemodynamics per 10 °C Increase in Ambient Temperature** | | | | |
| --- | --- | --- | --- | --- | --- |
|  | Main Model^a^ | Main Model Adjusted for PM_2.5_ | | Main Model Adjusted for Ozone | Main Model Adjusted for Dew Point |
| **Resting Blood Flow Velocity, cm/s** | | | |  |  |
| 1 | 0.5 (-2.9, 4.1) | 0.6 (-3.1, 4.5) | | 0.6 (-3, 4.3) | 2.2 (-2.9, 7.4) |
| 2 | -1.3 (-5, 2.5) | -0.3 (-4.5, 4) | | -0.9 (-4.8, 3.1) | 0.9 (-4.7, 6.8) |
| 3 | -2.2 (-6.2, 2) | -0.9 (-5.4, 3.9) | | -2 (-6.2, 2.5) | 0 (-6.2, 6.7) |
| 5 | -4.3 (-9, 0.5) | -3.3 (-8.4, 2.1) | | -4.1 (-8.9, 1) | -5.6 (-12.9, 2.2) |
| 7 | -4.6 (-9.8, 1) | -2.3 (-8.1, 3.8) | | -4.2 (-9.7, 1.6) | -4.6 (-13.3, 5) |
| 14 | -8.2 (-14.7, -1.3) | -5.6 (-12.7, 2) | | -8.4 (-15, -1.3) | -3.7 (-16, 10.4) |
| 21 | -10.1 (-17.3, -2.1) | -6.2 (-14, 2.4) | | -9.8 (-17.2, -1.8) | -16.2 (-29.5, -0.3) |
| 28 | -8.6 (-16.5, 0) | -4.2 (-12.8, 5.3) | | -8.7 (-16.6, 0) | -11.2 (-27.3, 8.6) |
| **Resting Cerebrovascular Resistance, mmHg.s/cm** | | | |  |  |
| 1 | -3.3 (-7.8, 1.4) | -3.9 (-8.7, 1.2) | | -3.5 (-8.2, 1.4) | -3.3 (-9.8, 3.6) |
| 2 | -2.3 (-7.2, 3) | -3.5 (-8.9, 2.3) | | -2.9 (-8.1, 2.6) | -2.3 (-9.7, 5.7) |
| 3 | -0.7 (-6.3, 5.2) | -2 (-8.1, 4.5) | | -1.1 (-7, 5.1) | -2.2 (-10.6, 7.1) |
| 5 | 2.4 (-4.5, 9.6) | 1.4 (-5.9, 9.3) | | 2.3 (-4.7, 9.8) | 2.2 (-8.5, 14.3) |
| 7 | 2.4 (-5.3, 10.8) | 0.9 (-7.2, 9.7) | | 2.4 (-5.6, 11) | 0.6 (-11.8, 14.8) |
| 14 | 9.3 (-1.2, 20.9) | 8 (-3, 20.2) | | 10.3 (-0.5, 22.3) | -6.4 (-22.4, 12.9) |
| 21 | 20.3 (7.1, 35.1) | 16.3 (3.1, 31.2) | | 20.3 (7, 35.3) | 12 (-11.7, 42.2) |
| 28 | 18.1 (4.3, 33.7) | 13.1 (-0.7, 28.7) | | 18.2 (4.3, 33.9) | 3.1 (-21.6, 35.4) |
| **Resting Mean Arterial Pressure, mmHg** | | | |  |  |
| 1 | -2.5 (-5.6, 0.6) | | -3 (-6.3, 0.4) | -2.6 (-5.8, 0.7) | -1.1 (-5.6, 3.6) |
| 2 | -3.3 (-6.6, 0.2) | | -3.5 (-7.2, 0.3) | -3.4 (-6.9, 0.2) | -1.3 (-6.4, 4) |
| 3 | -2.5 (-6.2, 1.4) | | -2.2 (-6.4, 2.1) | -2.4 (-6.4, 1.6) | -2.2 (-8, 4) |
| 5 | -1.1 (-5.6, 3.5) | | -0.7 (-5.6, 4.4) | -0.7 (-5.4, 4.2) | -3.4 (-10.5, 4.1) |
| 7 | -1.1 (-6.2, 4.2) | | -0.1 (-5.5, 5.7) | -0.5 (-5.8, 5) | -3.3 (-11.5, 5.6) |
| 14 | 1 (-5.6, 8.2) | | 3 (-4.2, 10.8) | 2.1 (-4.8, 9.5) | -10.4 (-21, 1.6) |
| 21 | 9 (0.7, 18) | | 10.6 (1.8, 20) | 9.5 (1.2, 18.6) | -6.2 (-20.2, 10.1) |
| 28 | 8.6 (-0.2, 18.2) | | 9.5 (0.3, 19.7) | 8.8 (-0.1, 18.4) | -8.5 (-23.8, 9.9) |
| **Cerebral CO_2_ Reactivity, cm/s.mmHg** | | | |  |  |
| 1 | 0.1 (-5.6, 6.1) | | -0.7 (-6.8, 5.8) | -0.4 (-6.2, 5.8) | 4.8 (-3.9, 14.3) |
| 2 | -1.2 (-7.3, 5.4) | | 0.1 (-6.8, 7.5) | -1.3 (-7.7, 5.6) | 6.1 (-3.8, 17) |
| 3 | 0.5 (-6.5, 7.9) | | 1.9 (-5.9, 10.4) | 0.1 (-7, 7.8) | 7.3 (-4.1, 20.1) |
| 5 | -3.1 (-10.9, 5.4) | | -2.2 (-10.8, 7.2) | -3 (-11, 5.8) | -1.8 (-14.5, 12.8) |
| 7 | -3.1 (-11.9, 6.6) | | -2 (-11.5, 8.5) | -4 (-13, 5.8) | 0.1 (-14.7, 17.6) |
| 14 | -9.1 (-19.5, 2.6) | | -7.8 (-18.8, 4.8) | -10 (-20.4, 1.7) | 8.2 (-13.8, 35.9) |
| 21 | -15.3 (-26.4, -2.7) | | -12.7 (-24.5, 0.9) | -15.4 (-26.5, -2.7) | -0.8 (-26.1, 33.2) |
| 28 | -13.6 (-25.6, 0.3) | | -11.3 (-24.1, 3.7) | -13.7 (-25.7, 0.2) | 2.9 (-26.3, 43.8) |

^a^ All models were adjusted for age, sex, race, smoking status, hypertension status, diabetes, body mass index, visit number, day of week, season, and long-term temporal trends.

**Table C.** Joint effects of ambient temperature and ozone on blood flow velocity.

| **Moving Average of Temperature and Ozone, day** | ***P* for Interaction**^a^ | **% Difference of Blood Flow Velocity per 10 °C Increase in Temperature** | |  | **% Difference of Blood Flow Velocity per 10 ppb Increase in Ozone** | |
| --- | --- | --- | --- | --- | --- | --- |
|  |  | Ozone at 25th percentile^b^ | Ozone at 75th percentile^b^ |  | Temperature at 25th percentile^c^ | Temperature at 75th percentile^c^ |
| 1 | 0.73 | 1.0 (-5.1, 7.4) | 0.6 ( -5.5, 7.0) |  | 0.2 (-3.3, 3.0) | -0.3 (-3.7, 3.4) |
| 2 | 0.59 | -0.3 (-6.9, 6.8) | -1.0 ( -7.5, 6.0) |  | 0.0 (-4.2, 2.9) | -0.8 (-4.8, 3.6) |
| 3 | 0.75 | -1.6 ( -8.6, 6.0) | -2.0 ( -9.0, 5.6) |  | 0.0 (-4.3, 3.6) | -0.5 (-4.7, 4.0) |
| 5 | 0.70 | -3.6 (-11.3, 4.8) | -4.2 (-11.8, 4.2) |  | -0.1 (-5.1, 3.7) | -0.8 (-5.7, 4.4) |
| 7 | 0.40 | -3.1 (-11.9, 6.6) | -4.5 (-13.2, 5.0) |  | 0.3 (-6.0, 3.7) | -1.4 (-7.4, 5.4) |
| 14 | 0.54 | -7.3 (-17.6, 4.3) | -8.4 (-18.6, 3.0) |  | 1.3 (-5.4, 5.6) | -0.1 (-6.6, 7.0) |
| 21 | 0.42 | -8.2 (-19.6, 4.7) | -9.8 (-21.0, 3.0) |  | -0.5 (-8.2, 3.9) | -2.5 (-9.8, 5.9) |
| 28 | 0.72 | -7.8 (-20.3, 6.5) | -8.6 (-20.9, 5.7) |  | 0.7 (-6.6, 6.6) | -0.2 (-7.3, 7.6) |

^a^ From models including covariates for temperature, ozone, the cross product of temperature and ozone, and adjusting for potential confounding factors.

^b^ The 25^th^ percentile for ozone raged from 16.0 to 18.0 ppb and the 75^th^ percentile range from 30.3 to 30.9 ppb.

^c^ The 25^th^ percentile for ambient temperature ranged from 2.81 to 3.38 °C and the 75^th^ percentile ranged from 18.1 to 18.7 °C.
